# Geographic origin and individual assignment of *Shorea platyclados* (Dipterocarpaceae) for forensic identification

**DOI:** 10.1371/journal.pone.0176158

**Published:** 2017-04-21

**Authors:** Chin Hong Ng, Soon Leong Lee, Lee Hong Tnah, Kevin Kit Siong Ng, Chai Ting Lee, Bibian Diway, Eyen Khoo

**Affiliations:** 1 Division of Forestry Biotechnology, Forest Research Institute Malaysia, Kepong, Selangor, Malaysia; 2 Sarawak Forestry Corporation, Kuching, Sarawak, Malaysia; 3 Forest Research Centre, Sandakan, Sabah, Malaysia; Consiglio Nazionale delle Ricerche, ITALY

## Abstract

The development of timber tracking methods based on genetic markers can provide scientific evidence to verify the origin of timber products and fulfill the growing requirement for sustainable forestry practices. In this study, the origin of an important Dark Red Meranti wood, *Shorea platyclados*, was studied by using the combination of seven chloroplast DNA and 15 short tandem repeats (STRs) markers. A total of 27 natural populations of *S*. *platyclados* were sampled throughout Malaysia to establish population level and individual level identification databases. A haplotype map was generated from chloroplast DNA sequencing for population identification, resulting in 29 multilocus haplotypes, based on 39 informative intraspecific variable sites. Subsequently, a DNA profiling database was developed from 15 STRs allowing for individual identification in Malaysia. Cluster analysis divided the 27 populations into two genetic clusters, corresponding to the region of Eastern and Western Malaysia. The conservativeness tests showed that the Malaysia database is conservative after removal of bias from population subdivision and sampling effects. Independent self-assignment tests correctly assigned individuals to the database in an overall 60.60−94.95% of cases for identified populations, and in 98.99−99.23% of cases for identified regions. Both the chloroplast DNA database and the STRs appear to be useful for tracking timber originating in Malaysia. Hence, this DNA-based method could serve as an effective addition tool to the existing forensic timber identification system for ensuring the sustainably management of this species into the future.

## Introduction

Degradation of forest resources leading to a loss of biodiversity is a global challenge. The 2015 Global Forest Resources Assessment performed by the Food and Agriculture Organization of the United Nations revealed that total global forest area has declined by 3%, from 4128 M ha in 1990 to 3999 M ha in 2015 [1]. Conversion of previously forested land to non-forest by both natural causes, e.g. drought, disease and fire, and human causes, e.g. clearance for agriculture, timber harvesting, the expansion of settlements, and infrastructure development have contributed to this decline [1]. Commercial logging activities have been reported to account for more than 70% of forest degradation in Latin America, the Caribbean and in Asia [2]. Apart from controlled logging activities, unseen illegal logging is also a major driving force behind deforestation and resulting in an estimated revenue loss of between USD 10 billion and 15 billion to the global market [3]. Revenue, which if invested in sustainable forest management could have safeguarded approximately 14 M ha of forest in Brazil; based on mitigation costs of USD 900/ha of avoided deforestation [4]. A number of international efforts have been initiated to combat deforestation and promote sustainable management of global forest resources. One of these is the development of forest certification scheme initiated by Forest Stewardship Council (FSC) in the early 1990s to ensure responsible management of the world’s forests and enable consumers to purchase timber that originates from sustainably managed forest [5, 6]. These efforts have produced encouraging results, for example there has been a 60% growth of forest managed according to FSC standards, from 118 M ha in 2009 to over 191 M ha in 2013[7].

Various initiatives have been implemented to prohibit the importation of any timber not obtained in accordance with the laws of the country of origin, e.g. Canadian Wild Animal and Plant Protection and Regulation of International and Interprovincial Trade Act (1992); US Lacey Act (amended 2008); EU Timber Regulation (2010); and Australian Illegal Logging Prohibition Act (2012) [8]. In order to fulfill the requirements of these regulations, the importers have to show the proof for species identity and origin of the wood concerned. In addition, demand for tracking systems is also highly called for as more consumers with sustainable awareness are willing to pay a premium for responsibly sourced certified wood. Consumer market demands therefore influence the logging activities. This evolution has driven the timber producer to improve the management on the flows of forest products and to ensure that forest products entering the timber markets are legally and sustainably produced.

A timber tracking system plays a role to fulfill the requirements of Chain of Custody certification and to gain knowledge of the supply chain structure. A key function of tracking systems is to trace the movement of timber material through the supply chain and this is achieved by product identification mechanisms which are able to link the physical wood or wood products to the database developed for respective timber species [9]. Current timber identification methods vary in different degree of complexity, depending on the affordable costs and availability of technology. The methods for identifying timber have been reviewed [8] and these can be divided into three categories, namely (1) visual methods, e.g. wood anatomy: internal structure of wood at the macroscopic and microscopic level [10] and dendrochronology: the study of periodic growth increments formed in most temperate tree species [11]; (2) chemical methods, e.g. mass spectrometry: assessment of phytochemicals present in the heartwood [12], near infrared spectroscopy: the characterizations of wood absorption spectra [13], stable isotopes: chemical compounds synthesized by trees such as carbon, hydrogen, oxygen, nitrogen [14] and radiocarbon dating: the calculation of radiocarbon age based on the ratio ^14^C and ^12^C atoms [15]; (3) genetic methods: providing species level identification based on variation at specific gene regions [16], geographic origin identification using population genetics and phylogeographic analyses [17, 18] and individual level identification utilizing DNA profiles generated from molecular markers which can reveal variations between individuals [19–21].

Each of the tracking system has its own advantages and shortcomings [8, 22]. The cost saving methods for documenting wood origin are based on non-inherent features of wood such as painted identification marks, plastic tags or the use of paper-based certification, where the information is passed along the supply chain together with the raw material. This system however, is vulnerable to falsification. In order to overcome this problem, methods based on inherent wood characteristics such as the unique properties of DNA, can provide unambiguous validation of the chain-of-custody documentation. The use of DNA is resistant to forgery and samples can be taken at any stage in the supply chain. As an addition to the current system, it enables the accuracy of existing documents to be verified, which makes the timber trade more transparent. Molecular DNA technology in timber tracking has expanded tremendously in the last decade due to technological advances made in the field of human forensic genetics, which has facilitated application transfer to various non-human species, including plants.

The application of DNA markers in timber tracking has been reported in geographic origin assignment [23, 24] and individual assignment [19, 21]. Geographic origin assignment is based on the ability to assign a sample to a particular population, requiring the source population to be sufficiently genetically distinct from other candidate populations and relying on the existence of population data from multiple areas [23]. The population data are transformed into genographic map representing spatial genetic structure observed at both local and regional scales of a species [25, 26]. These population identification maps have been used to verify the origins of timbers, starting with an traceability system to differentiate the western versus eastern *Quercus* populations in Europe [27]. Similar positive results were achieved for population assignment of *Neobalanocarpus heimii*, a dipterocarp timber species, to northern and southern regions of Peninsular Malaysia [24]. In addition, successful assignment has also been demonstrated in a CITES listed species, *Gonystylus bancanus*, to support forensic applications and help safeguard the species [19]. Apart from geographic origin assignment, DNA based verification can also function at the individual level. If DNA samples are taken from each standing tree prior to felling, a unique DNA profile can be generated per log, which can be verified at each step along the supply chain [28]. By genotyping a broad enough set of reference database, it is possible to calculate the random match probability or the estimated frequency at which a particular STR profile would be expected to occur in a population [29] This random match probability is usually very small, e.g. goes beyond one in billions (10^9^) [19] or trillions (10^12^) [21], leading to the acceptance of DNA profiling as admissible evidence in court cases, where legal requirements are met. The DNA profiling technology has been used to verify intact chain of custody for *Intsia palembanica* in Indonesia [28], by matching samples taken at different points as they pass through the supply chain. In addition, the existing individual identification database developed for *N*. *heimii* [21] and *G*. *bancanus* [19] present opportunities for authority personnel to link wood material seized from suspects with the stumps of illegally felled trees.

In this study, we used a suite of chloroplast markers to develop population identification database and STR markers to develop individual identification database capable of resolving geographic origin and individual identity of *Shorea platyclados* (Dipterocarpaceae). *S*. *platyclados* (Dipterocarpaceae) is locally known as Meranti bukit and can be found in hilly and mountainous areas of Peninsular Malaysia, Borneo and Sumatra [30]. It is confined to the upper dipterocarp forest zone and inhibits ridges, valleys and hillsides with altitudinal range between 800 and 1000 m. Typically high(er) altitude forest has been avoided by loggers, but the threat in these areas is increasing due to depletion of more lowland areas. Threat level in Malaysia for this species has been classified as “near threatened” in Malaysia [31]. The timber is traded as Dark Red Meranti in the international market where it has been used as a popular general utility timber for furniture manufacture, fancy doors, high class interior finishing, flooring, panelling and partitioning [32]. Owing to the high demand for this timber, and with the new timber regulations in international markets, it is timely to strengthen the existing timber tracking system, and make it more robust and recognizable. Therefore, we aimed to 1) generate a population identification database of *S*. *platyclados* for geographic origin identification; 2) generate a DNA profiling database for individual identification of *S*. *platyclados* based on a subpopulation-cum-inbreeding model; 3) evaluate the reliability of the individual identification database using Bayesian assignment approaches.

## Material and methods

### Population sampling and DNA extraction

A total of 27 populations representing its Malaysian distribution of *S*. *platyclados* were sampled. These populations were selected based on the record and site survey information provided by the Forest Department. The individuals within the populations were sampled randomly. The permission to access the forest has been granted by Forest Department in respective state. The locations of sampled populations are shown in Table 1. The samples were collected either in the form of inner bark or leaf tissues. Total genomic DNA was extracted following a modified CTAB method [33], and purified using a High Pure PCR Template Preparation Kit (Roche Diagnostics GmbH). The purified DNA was used in both chloroplast DNA and STR analysis. A subset of the samples comprising 214 individuals was used for chloroplast DNA analysis between six and eight samples per population with the exception of six samples used in Hulu Terengganu Forest Reserve (Table 1). For STRs analysis, all the collected samples with total number of 1032 individuals were used (Table 1).

**Table 1:** Geographic origin and sample size (*N*) for both identification databases, population (Pop) and individual (Ind), for the 27 Malaysian populations included in this study.

Population	Code	State of origin	Location	*N*	
				Pop	Ind
Petuang	1	Terengganu	N5.34 E102.71	8	16
Tembat	2	Terengganu	N5.19 E102.60	8	47
Hulu Terengganu	3	Terengganu	N4.95 E102.92	6	6
Gunung Basur	4	Kelantan	N5.51 E101.81	8	35
Balah	5	Kelantan	N5.25 E101.63	8	19
Gunung Stong	6	Kelantan	N5.21 E101.95	8	36
Sungai Betis	7	Kelantan	N4.75 E101.44	8	37
Gunung Rabong	8	Kelantan	N4.83 E102.14	8	35
Belum	9	Perak	N5.61 E101.66	8	32
Temenggor	10	Perak	N5.52 E101.61	8	36
Piah	11	Perak	N5.09 E101.33	8	29
Bintang Hijau	12	Perak	N4.93 E100.88	8	44
Bukit Larut	13	Perak	N4.87 E100.79	8	75
Bubu	14	Perak	N4.66 E100.84	8	11
Bukit Kinta	15	Perak	N4.53 E101.30	8	25
Bukit Tapah	16	Perak	N4.40 E101.38	8	95
Ulu Jelai	17	Pahang	N4.61 E101.90	8	86
Fraser	18	Pahang	N3.70 E101.73	8	45
Awana	19	Pahang	N3.39 E101.79	8	46
Gunung Bunga Buah	20	Pahang	N3.37 E101.77	8	31
Bukit Tinggi	21	Pahang	N3.35 E101.79	8	41
Lentang	22	Pahang	N3.24 E102.03	8	10
Semangkok	23	Selangor	N3.64 E101.74	8	52
Berembun	24	N. Sembilan	N2.83 E102.05	8	44
Nanga Amang	25	Sarawak	N1.51 E113.09	8	41
Putai	26	Sarawak	N1.52 E114.55	8	29
Rafflesia	27	Sabah	N5.77 E116.35	8	29

### Population identification database

Universal primers for 27 chloroplast markers [34] were screened against eight individual of *S*. *platyclados* from across the species’ range for their ability to amplify a product. Seven non-coding chloroplast DNA regions were found to be informative for the characterization of haplotypes in *S*. *platyclados*: the intergenic spacers of *trn*T-*trn*L, *trn*S-*trn*G, *atp*B-*rbc*L, *pet*G-*trn*P, *trn*G-*atp*A, *psb*M-*trn*D and *trn*G-*rps*14 (S1 Table). PCRs were performed in 10 μL reaction volumes containing 1x Type-it Multiplex PCR Master Mix (Qiagen), 0.2 μM of each primer and 10 ng of template DNA. The reactions were carried out using an Applied Biosystems 2720 Thermal Cycler with the following reaction conditions: initial activation step at 95°C for 5 min, 35 cycles of 95°C for 30 s, 50°C for 90 s, 72°C for 1 min and a final extension at 60°C for 30 min. PCR products were purified using ExoSAP-IT reagent (GE Healthcare) and sequenced in both directions using an ABI 3130xl Genetic Analyzer (Applied Biosystems). Sequencing data were edited and assembled using Sequencher 5.1 (Gene Codes Corporation), and submitted to GenBank under accession numbers KU170146-KU170186. Haplotypes were determined from nucleotide substitutions and indels (insertion and deletions).

Chloroplast DNA is maternally inherited and expected to be highly geographically structured by showing large proportion of population specific haplotypes. A unique haplotype was defined as one found in a single population only. These haplotypes were defined based on the 214 sample dataset collected from 27 populations and used to generate a haplotype distribution map throughout Malaysia. The population identification database was constructed as reference to classify the samples into their population of origin based on the significant intraspecific variable sites within the chloroplast regions.

### Individual identification database

#### STR genotyping and reproducibility

A total of 15 STR loci developed specifically for *S*. *platyclados* were used for genotyping all the samples: *Spl003* [35], *Spl529*, *Spl599*, *Spl600*, *Spl629*, *Spl667*, *Spl676*, *Spl690*, *Spl763*, *Spl764*, *Spl834*, *Spl845*, *Spl855*, *Spl858* and *Spl863* [36]. The PCR amplifications were performed in 8 μl reactions volumes containing 10 ng of template DNA, 1x Type-it Multiplex PCR Master Mix (Qiagen) and 0.4 μM of each forward and reverse primer. The labelled forward primer was diluted with non-labelled primer with the ratio 1:10. This dilution step enables the PCR product to be used directly in fragment analysis without further dilution to reduce the intensity of the fluorescent signal. The PCR amplification was performed in an Applied Biosystems 2720 Thermal Cycler using the following reaction conditions: initial activation step at 95°C for 5 min, 35 cycles of 95°C for 30 s, 50°C for 90 s, 72°C for 30 s and a final extension at 60°C for 30 min. The PCR products were electrophoretically separated using an ABI 3130xl Genetic Analyzer (Applied Biosystems). Allele sizes of the samples were assigned against the ROX 400HD internal size standard (Applied Biosystems) using Genemapper v4.0 software (Applied Biosystems). Reproducibility was tested by performing five independent PCR amplifications on one individual and comparing the resulting genotype data [37].

#### Population clustering

The population structure of *S*. *platyclados* in Malaysia (27 populations) was inferred based on a model-based clustering method using the program Structure v2.3.4 [38]. All the 1032 samples were assigned probabilistically to *K* populations using Markov chain Monte Carlo (MCMC) method without using predefined population information. The run consists of a burn-in length of 250,000 iterations before collecting data to minimize the effect of the starting configuration. Subsequently, the simulation was run for 850,000 iterations, assuming an admixture model and correlated allele frequencies. The default program was applied for other settings and no prior information was used to define the groups. Three independent runs of each *K* between 1 and 6 were performed to produce consistent results. The results were analysed by the online version of Structure Harvester [39] and the most likely number of groups (*K*) was chosen based on the Delta *K* statistic [40].

#### Characterizations of database

The characterization of the database was carried out for 1032 individuals in Malaysia. The allele frequency of each locus was calculated using Microsatellite Toolkit [41]. The number of alleles per locus (*A*), observed heterozygosity (*H*_o_) and expected heterozygosity (*H*_e_) were calculated using Genetic Data Analysis (GDA) version 1.0 [42]. The effectiveness of the STR loci was assessed by calculating the polymorphic information content (PIC), matching probability (MP) and power of discrimination (PD) using the PowerStats v.1.2 spread sheet (Promega Corporation, USA) as described [43]. The evaluations of Hardy-Weinberg Equilibrium (HWE) and Linkage Equilibrium (LE) were performed using the Exact Test in GDA. A sequential Bonferroni correction was used to compensate for multiple comparisons among loci [44]. The coancestry coefficient (*θ*) and inbreeding coefficient (*f*) were calculated using GDA and subjected to bootstrapping of 1000 replicates across loci for significant testing [45]; using samples from three hierarchical level: Malaysia, Western Malaysia and Eastern Malaysia. These two values *θ* and *f* will be used to correct the calculation of profile frequency as the STR loci showed deviation from HWE and LE in the Malaysian database.

#### Conservativeness of database

The profile frequency or random match probability was calculated using the subpopulation-cum-inbreeding model [46] for the individuals from Malaysian database. The conservativeness of the database was calculated using only samples with no missing data. Owing to small sample size (<16 individuals), populations from Hulu Terengganu, Bubu and Lentang were omitted from the analysis. The minimum allele frequency was adjusted to 5/(2*N*) where *N* is the number of individuals sampled from Malaysian database; to enable each allele be observed at least five times in total to be included in reliable statistical calculation [47]. The profile frequency is calculated by first considering the genotype frequency for each locus and then multiplication the frequencies across all loci, while the random match probability is the reciprocal of it [29]. The conservativeness of the Malaysian database was determined by calculating the full profile frequency of each region (Western Malaysia and Eastern Malaysia) tested on its own data set (*P*_origin_) and the full profile frequency of each region tested against the Malaysian database (*P*_combined_). The differences (*d*) between the two were defined as: *d* = log_10_(*P*_origin_/*P*_combined_). A database will be conservative if *P*_origin_ was less than *P*_combined_ and indicated by negative *d* value [48]. Database robustness was *θ* adjusted to overcome the problem of subpopulation that is possibly not represented in the data set [48]. A series of *θ* were applied to adjust the profile frequency, of which *P*_combined_ was recalculated and *P*_origin_ remain unchanged. The effectiveness of *θ* adjustment was observed by calculating mean *d* and proportion of samples carrying negative *d* values.

#### Assignment test

In order to test the performance of the *S*. *platyclados* database (Malaysian database) in inferring the origin of any individual, we performed two independent self-assignment tests. Firstly, by the existing designated 24 populations; and secondly, by regions (Western and Eastern Malaysia). Three populations, Hulu Terengganu, Bubu and Lentang, were excluded from this analysis due to small sample size (< 16 individuals), with potential problems of low correctly assignment rate. The individual’s multilocus STR genotype of the reference data were self-classified to each population or region using the Bayesian multilocus approach [49] in Geneclass2 [50].

#### Application of the Malaysian database

We have carried out sampling on a batch of *S*. *platyclados* which were planted on the campus of the Forest Research Institute Malaysia (FRIM) (N3.23 E101.63). The sampling was aim to trace the origin of the planted *S*. *platyclados* by using the proposed timber tracking method developed for the species itself. A total of 40 individuals were sampled for DNA extraction applying the same methodology as mentioned above. Total genomic DNA was extracted from inner bark tissues. The samples were screened for the seven selected chloroplast DNA markers, and genotyped for the 15 STRs loci following similar protocol as described earlier. Subsequently, the generated *S*. *platyclados* database from the current study was used to trace the origin of the planted *S*. *platyclados* in two scenarios: (1) to reveal the source of origin from Western or Eastern Malaysia using population identification database, (2) to assign the sample to its source of origin population with multilocus STR genotypes assignment test using individual identification database.

## Results

### Population identification database

**Fig 1:**
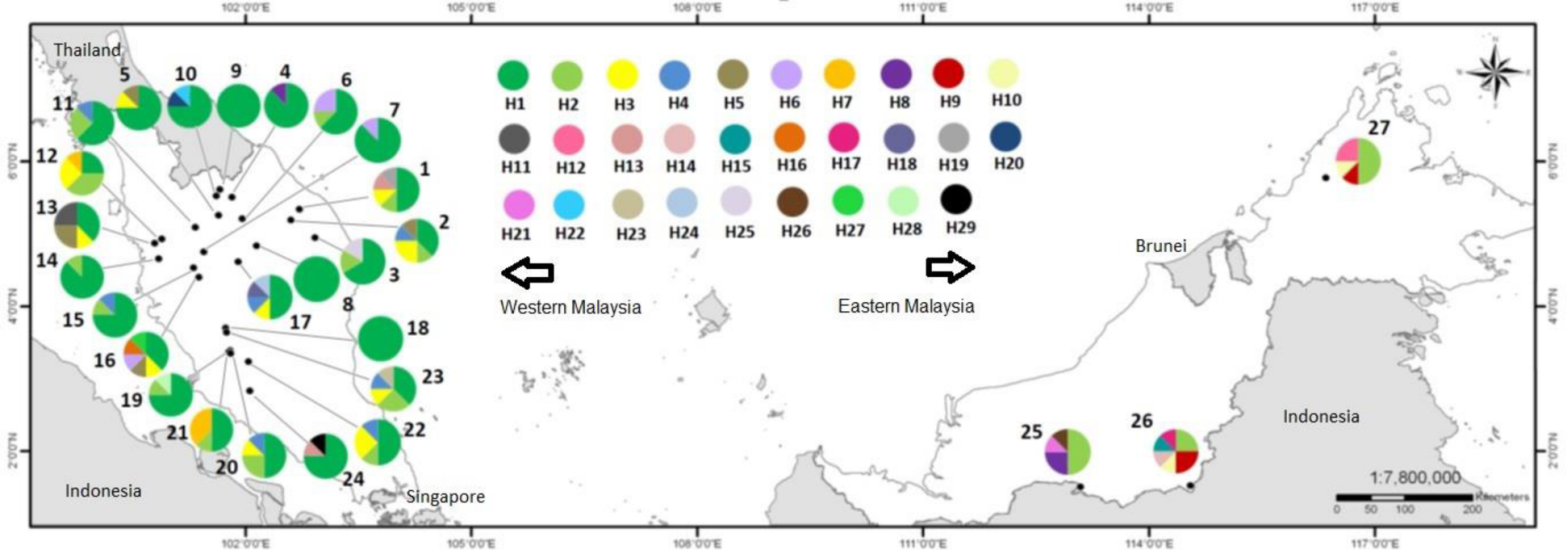
A distribution map showing the geographic locations of *Shorea platyclados* population (1–27), and the distribution of haplotypes (H1 –H29) across these populations in population identification database. The pie chart size corresponds to the sample size at each location.

Of the seven variable and informative chloroplast DNA markers sequenced, we resolved sequences of the following lengths: *trn*T-*trn*L (314 bp), *trn*S-*trn*G (277 bp), *atp*B-*rbc*L (531 bp), *pet*G-*trn*P (449 bp), *trn*G-*atp*A (362 bp), *psb*M-*trn*D (559 bp) and *trn*G-*rps*14 (400 bp). There were 31 base substitutions and eight indels across the combined intergenic regions (S2 Table). Among these variable sites, two were found in *trn*T-*trn*L, eight in *trn*S-*trn*G, five in *atp*B-*rbc*L, three in *pet*G-*trn*P, five in *trn*G-*atp*A, 11 in *psb*M-*trn*D and five in *trn*G-*rps*14. A total of 29 haplotypes were defined based on these variable sites, 16 of which, occurred in single individuals (H14 –H29, Fig 1).

The most common haplotype, H1 was present in 57.5% of the samples, and was confined to Western Malaysia. This haplotype was geographically widespread throughout Western Malaysia, and is represented in each sampled population within the region. In three populations (Gunung Rabong, Belum and Fraser) the individuals sampled all contained this haplotype. The second most common, H2 was found in 13.1% of the sampled individuals, and was present in both Western and Eastern Malaysia. Only one other haplotype was present in both regions, H8 which was found in 1.4% of individuals. The remaining haplotypes were confined to either Western (H3-H7, H11, H13, H16, H18-H20, H22-H25, H27-H29) or Eastern (H9, H10, H12, H14, H15, H17, H21, and H26) Malaysia. Based on the chloroplast DNA haplotypes distributions, the results suggested population which carries haplotype H1 will be most likely originated from Western Malaysia. If haplotype H9, H10 or H12 were to be revealed in populations, the populations could be inferred back to be originated from Eastern Malaysia. These three haplotypes were not found in Western Malaysia.

### Individual identification database

#### Reproducibility of the STR markers

The 15 STRs gave consistent genotypes for the five independent amplifications, with *d*-values (the base pair size different between minimum and maximum fragment lengths) less than 0.5 in all cases.

#### Population clustering

**Fig 2:**
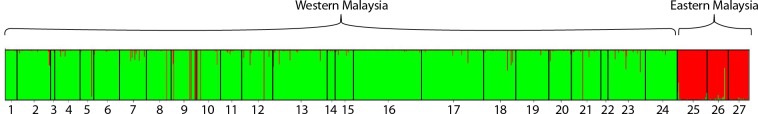
Bayesian clustering (structure) based on 15 loci. **Individual posterior probabilities for inferred number of clusters (*K* = 2, see S1 Fig for justification).** Green and red bars indicate the individual posterior probability of belonging to cluster 1 and 2, respectively, and represent the geographic split between Western and Eastern Malaysia. The population codes (1–27) are listed below the clusters.

The Structure Harvester produced the highest likelihood scores when the value of *K* was set at 2 based on Delta *K* statistic (S1 Fig), and all the 1032 individuals could be assigned to one of the two genetic clusters (Fig 2). The green cluster represents the populations located in Western Malaysia and the red cluster belongs to populations located in Eastern Malaysia although there is evidence in several populations of admixture. These two regions are separated by the South China Sea, probably a barrier to limit the exchange of genetic materials among them.

#### Characterization of Malaysian database

Each of the individual in the Malaysian database carries a unique STR multilocus genotype. The distributions of allele frequencies for Malaysia are shown in S3 Table. In order to have a reliable statistical calculation, the minimum allele frequency was adjusted to 0.0024 for Malaysia. The characterizations of genetic diversity and forensic parameters are shown in Table 2. A total of 303 alleles (*A*) were observed among the 15 STR loci in Malaysia with the minimum of 12 alleles in *Spl*600 and maximum of 31 alleles in *Spl*676 (Table 2). The power of discrimination (PD) shown by the STR markers ranged from 0.7570 (*Spl*690) to 0.9800 (*Spl*676). The *Spl*676 contained the maximum number of alleles and was the most discriminating locus among the 15 loci tested. However, the minimum number of alleles in *Spl*600 (*A* = 12; PD = 0.8170) did not lead this marker to be the poorest discriminator; the marker with lowest PD was *Spl*690 (*A* = 19; PD = 0.7570). The power of discrimination is determined by the characteristics of the allele represented by the marker and not solely on the numbers of alleles.

**Table 2:** Genetic diversity and forensic variables for each the 15 STR loci of *Shorea platyclados* in the individual identification database.

	*Spl*003	*Spl*529	*Spl*599	*Spl*600	*Spl*629	*Spl*667	*Spl*676	*Spl*690	* Spl*763	* Spl*764	* Spl*834	* Spl*845	*Spl*855	*Spl*858	*Spl*863
*A*	17	16	13	12	21	26	31	19	18	23	17	28	19	25	18
*H*_*o*_	0.7583	0.5121	0.7198	0.5998	0.5451	0.8464	0.8717	0.5306	0.6298	0.5981	0.7820	0.7746	0.5300	0.8391	0.5354
*H*_*e*_	0.7995	0.5826	0.7533	0.6467	0.6107	0.8633	0.8973	0.5799	0.6902	0.6513	0.8425	0.7998	0.6322	0.8602	0.5807
PIC	0.7700	0.5100	0.7200	0.5900	0.5800	0.8500	0.8900	0.5100	0.6500	0.6200	0.8200	0.7800	0.5800	0.8500	0.5100
HWE	0.0000^b^	0.0000^b^	0.0006^b^	0.0000^b^	0.0000^b^	0.0000^b^	0.0003^b^	0.0000^b^	0.0000^b^	0.0000^b^	0.0000^b^	0.0000^b^	0.0000^b^	0.0000^b^	0.0000^b^
MP	0.0680	0.2380	0.0970	0.1830	0.1950	0.0340	0.0200	0.2430	0.1580	0.1560	0.0430	0.0650	0.1830	0.0360	0.2420
PD	0.9320	0.7620	0.9030	0.8170	0.8050	0.9660	0.9800	0.7570	0.8420	0.8440	0.9570	0.9350	0.8170	0.9640	0.7580

*A*: Total number of alleles; *H*_o_: Observed heterozygosity; *H*_e_: Expected heterozygosity; PIC: Polymorphic information content; HWE: Hardy-Weinberg equilibrium; MP: Matching probability; PD: Power of discrimination.

^b^ Significant deviations from HWE after Bonferroni adjustment (*P* < 0.05/15 = 0.0033).

Each of the 15 tested STRs deviated significantly from HWE evaluation after Bonferroni adjustment. Subsequently, we evaluated the STRs in each population independently to rule out the genotyping errors or the present of null alleles which caused the markers for showing deviations from HWE. The observed deviations from HWE were ranged from 6.7% (one locus) to 33.3% (five loci) in six populations; namely Tembat, Hulu Terengganu, Belum, Lentang, Putai and Rafflesia (S4 Table); and these deviations were lower compared to the entire Malaysian database. The STR loci in the remaining 21 populations were at HWE (S4 Table). This suggests that the significant deviation from HWE observed in Malaysian database might due to the Wahlund effect caused by population substructuring of the split between Western and Eastern Malaysia. The LE tests showed that all 105 pairwise comparisons between each allele were significant after employing Bonferroni corrections for multiple comparisons in Malaysian database. Likewise, the LE tests were also evaluated in each population independently (S4 Table). The results showed a range of 1.9% to 19.0% of the 105 pairwise loci comparisons were significant after Bonferroni adjustment in 24 populations (S4 Table). The STR markers were not linked in three populations namely, Bukit Tapah, Ulu Jelai and Awana. Therefore, the deviations from the independence test in Malaysian database might due to population substructuring as observed in the HWE evaluation.

The *θ* and *f* values for each of the hierarchical levels were significantly greater than zero, as the 95% confidence interval did not overlap with zero (Table 3). The *θ* value in the total Malaysian sample (0.0603) was higher than in Western Malaysia (0.0333) but lower than Eastern Malaysia (0.1064), suggesting that 3.33% and 10.64% of the genetic variability was distributed among populations in Western and Eastern Malaysia respectively. Similarly, the *f* value in the total Malaysian sample (0.0665) was also greater than in Western Malaysia (0.0358) but lower than Eastern Malaysia (0.1248).

**Table 3:** Coancestry coefficients (θ) and inbreeding coefficients (f) of *Shorea platyclados* calculated according to hierarchical levels. Probability of the mean θ and f were determined using bootstrap analysis (1000 replications) and results were presented with 95% confidence interval. Sample size (*N*) is given in parentheses.

Hierarchical level	Coancestry coefficient (*θ)*			Inbreeding coefficient (*f)*		
	Mean	2.5%	97.5%	Mean	2.5%	97.5%
Malaysia (*N* = 1032)	0.0603	0.0498	0.0736	0.0665	0.0501	0.0877
Western Malaysia (*N* = 933)	0.0333	0.0295	0.0373	0.0358	0.0258	0.0499
Eastern Malaysia (*N* = 99)	0.1064	0.0751	0.1466	0.1248	0.0857	0.1744

#### Conservativeness of database

**Fig 3:**
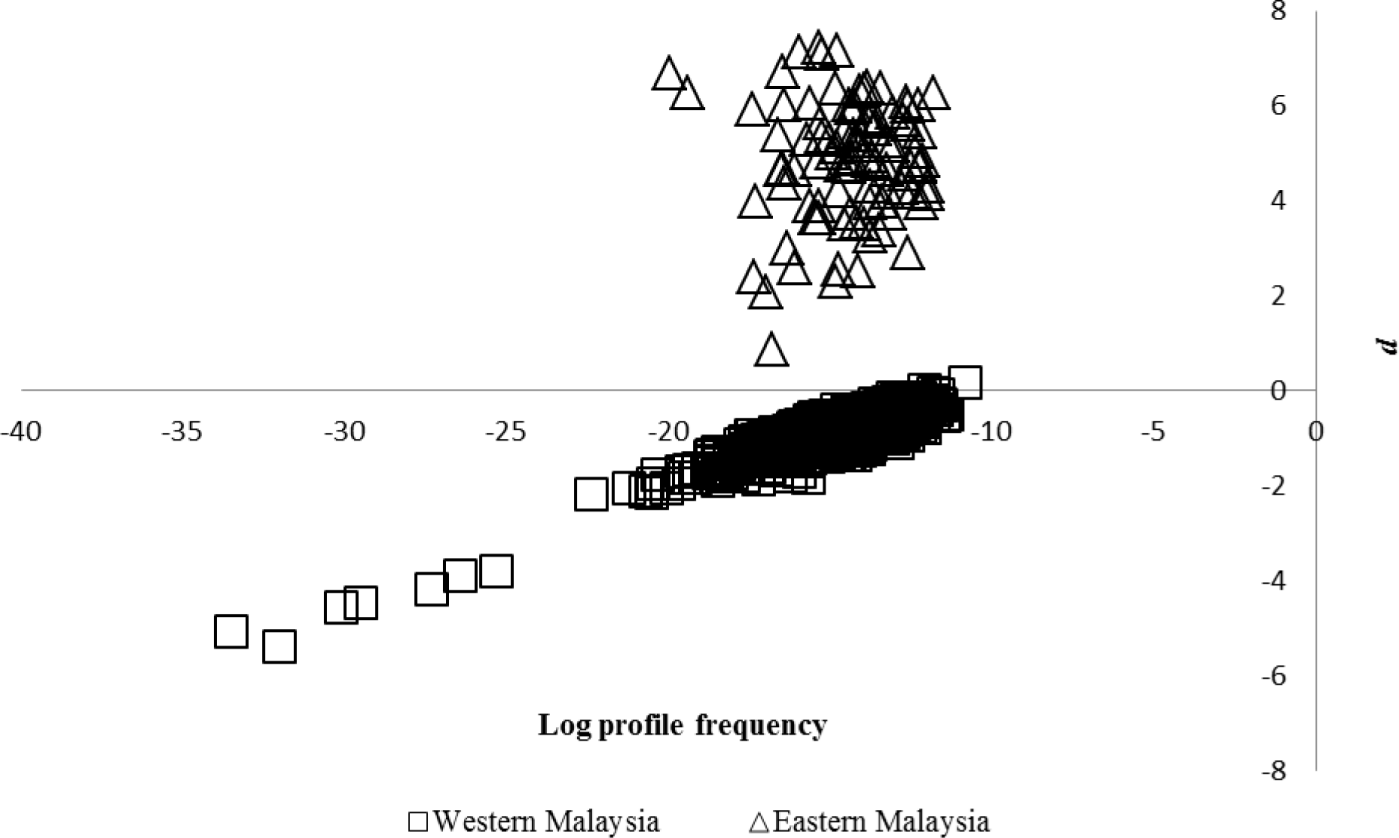
The conservativeness of the Malaysian database tested by estimating the parameter *d* for every DNA profile frequency using the subpopulation-cum-inbreeding model. X-axis: log profile frequency and Y-axis: mean number of differences between origin and combined database (*d*).

**Fig 4:**
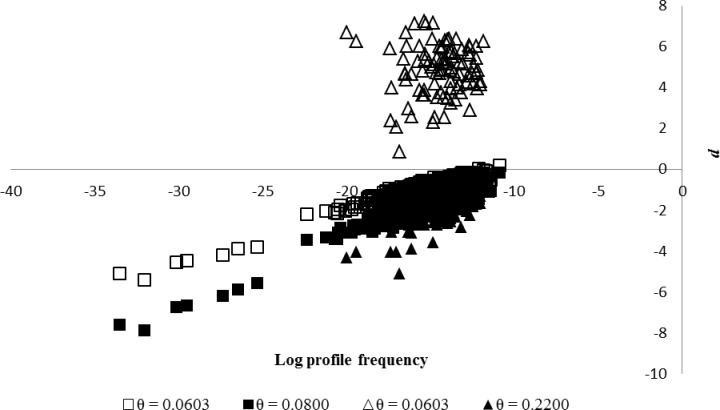
The effect of applying coancestry coefficients (*θ*) adjustment to the conservativeness of Malaysian database based on Western (square) and Eastern Malaysia (triangle) regions when tested by estimating the parameter d for every DNA profile frequency in the database. X-axis: log profile frequency and Y-axis: mean number of differences between origin and combined database (d).

The conservativeness of the Malaysian database was calculated based on full profile frequency in Western (911 individuals) and Eastern Malaysia (95 individuals) tested on its own dataset (*P*_origin_) and the full profile frequency of each region tested against the Malaysian database (*P*_combined_). A total of 909 tests performed on Western Malaysia showed negative *d* values, indicating 99.8% of the Malaysian database was conservative at the calculated *θ* value of 0.0603 (Fig 3). There was a slight tendency for *P*_origin_ > *P*_combined_ at *θ* = 0.0603 in Western Malaysia and this lead to non-conservativeness of the Malaysian database. This bias could be removed by gradual incrementation from *θ =* 0.0603 to *θ =* 0.0700 and subsequently to *θ* = 0.0800 (Fig 4). All the 911 tests performed on Western Malaysia were conservative when the *θ* was adjusted to 0.0800. Therefore, the *θ* value of 0.0800 will be used in Western Malaysia to ensure the 100% conservativeness of the Malaysian database. This adjustment to *θ* resulted in an increase in the overall mean profile frequency from 2.5400x10^-13^ (*θ* = 0.0603) to 9.2693x10^-13^ (*θ* = 0.0800) in Western Malaysia. For the Eastern Malaysia, all the 95 tests performed showed non-conservativeness at the calculated *θ* value of 0.0603 (Fig 3). To ensure conservativeness, *θ* values were incrementally increased (in increments of 0.01) until conservativeness across all tests in the region was achieved. This resulted in a *θ* = 0.2200, and a mean profile frequency changed from 6.9000x10^-18^ (*θ* = 0.0603) to 3.9100x10^-12^ (*θ* = 0.2200) (Fig 4). Therefore, the *θ* will be adjusted from 0.0603 to 0.0800 in Western Malaysia and to 0.2200 in Eastern Malaysia to ensure the conservativeness of Malaysian database.

#### Assignment test

In order to test the performance of the Malaysian database, a self-assignment test was performed for individuals to infer back the source of origin to each *S*. *platyclados* within the population and region. The overall correct assignment rate ranged from 60.60% (Western Malaysia) to 94.95% (Eastern Malaysia) in identified populations and 98.99−99.23% in identified regions respectively (Table 4). The assignment rate based on region was over 98.00% and relatively higher than in individual populations.

**Table 4:** The proportion of *Shorea platyclados* individuals correctly assigned to both population and region using self-assignment test.

Region	Population	Correctly assigned individuals	
		By population	By region
Western	Petuang	25.00%	99.23%
Malaysia	Tembat	55.32%	
	Gunung Basur	68.57%	
	Balah	31.58%	
	Gunung Stong	47.22%	
	Sungai Betis	70.27%	
	Gunung Rabong	82.86%	
	Belum	65.63%	
	Temenggor	44.44%	
	Piah	68.97%	
	Bintang Hijau	59.09%	
	Bukit Larut	62.67%	
	Bukit Kinta	48.00%	
	Bukit Tapah	68.42%	
	Ulu Jelai	60.47%	
	Fraser	62.22%	
	Awana	67.39%	
	Gunung Bunga Buah	61.29%	
	Bukit Tinggi	58.54%	
	Semangkok	59.62%	
	Berembun	56.82%	
	**Overall**	**60.60%**	
Eastern	Nanga Amang	95.12%	98.99%
Malaysia	Putai	93.10%	
	Rafflesia	96.55%	
	**Overall**	**94.95%**	

Type I error = 0.05.

#### Application of the Malaysian database

The population identification database developed in this study was used to infer the geographic origin of *S*. *platyclados* located within the grounds of FRIM. Of the 40 individuals screened, 67.5% of the samples contained haplotype H1, 10.0% contained H2, 5.0% contained H3, 2.5% contained H5 and the remaining 15.0% were haplotypes previously not identified. As haplotype H1, H3 and H5 are confined to Western Malaysia; these results suggested that 75.0% of the planted *S*. *platyclados* likely originated in this region.

In order to narrow down the origin of the planted individual to specific populations, we applied assignment test based on the individual identification database. The assignment test suggested an origin for 14 samples, each with confidence scores over 80%. Among the samples, seven of the individuals were assigned to Bintang Hijau, five to Temenggor, one each to Bukit Larut and Bukit Kinta. These four populations are in close geographic proximity and are located in the northwest of Western Malaysia. Therefore, the seed source for the *S*. *platyclados* planted in FRIM might come from northwest of Western Malaysia.

## Discussion

This study presented identification databases for an important Dark Red Meranti wood *S*. *platyclados* in Malaysia at two hierarchical levels, population and individual. These databases were validated by locating the origin of *S*. *platyclados*, which are located on the FRIM campus. The first, at the population level, using seven chloroplast DNA. The second, at the individual level, using 15 STR markers which are expected to reveal patterns formed by the combine effects of gene flow, mutation and random genetic drift [51]. These markers will allow the user to judge the origin of a sample based on shared polymorphism with individuals from a given population. Chloroplast markers and STRs are generally utilized differently. For example, chloroplast DNA is generally maternally inherited in angiosperms thus is transmitted via seeds only, leading to variation in this region being highly geographically structured [52]. Specific haplotypes are thus inherited unchanged through generations although it subjected to mutation. A study on chloroplast DNA variation in European *Quercus* based on data from over 2600 populations revealed strong geographic structuring along an east-west divide [25, 53, 54]. In addition, a structure divide between east-west was also observed in populations of *G*. *bancanus* from Malaysia [19]. In contrast, nuclear STR markers are bi-parentally inherited and genes can be dispersed through both pollen and seeds. STR markers tend to be used in individual assignment when a higher degree of resolution is required, for example in timber tracking. Genetic assignment methods can be applied to test if the genotype of a specific individual is likely to originate from a given population. The greater the number of markers used, the less likely it is that another individual has the same series of alleles. Therefore, a representative sample of DNA profiles from the population has to be established for statistical evaluation on the probability of a random match. Two samples having a similar profile may indicate shared heritage but the probability that this happen by chance needs to be considered.

In this study, the population identification database was established using seven chloroplast DNA regions. The combined chloroplast DNA regions increased the power of discrimination to overcome the variability problem caused by maternally inherited DNA markers. The sampling strategy employed in this study covered much of the distribution range of *S*. *platyclados* in Western Malaysia; however sampling density in Eastern Malaysia was smaller. The sampling density per unit area is different as Eastern Malaysia is under-represented in the sampling design. Based on shared common haplotypes, the resolution of our seven chloroplast DNA markers is not high enough to trace unknown samples to individual forest reserves, which are classified based on human political boundaries. It is possible to infer the geographic origin of *S*. *platyclados* at the regional level from this data (i.e. either from Western or Eastern Malaysia). An unknown sample carrying one of the 19 haplotypes confined to Western Malaysia will infer the geographic origin from this region. Similarly, a second unknown sample carrying one of the eight haplotypes confined to Eastern Malaysia will infer the geographic origin from that region.

The present population identification database could be useful for forensic timber identification in two circumstances. The first is to verify whether a sample is confirmed from the announced geographic region of Western or Eastern Malaysia. The second is to exclude a given origin if the unknown sample does not carry a haplotype in the database. While this exclusion testing would not possible with the current population level database, it may be possible with exhaustive sampling of individuals from all populations. The current population database could be continuously updated with more sampling sites incorporated, especially in Eastern Malaysia. This is essential for the accurate use of the database as assigning an individual to Eastern Malaysia is currently very unlikely. Apparently more effort has to be carried out to increase the comprehensiveness of the database for chain of custody certifications, by increasing the number of unique haplotypes in the database.

If an unknown sample has been traced back to Western or East Malaysia, it is then theoretically possible to trace individual origin using the individual identification database. One of the important things in considering an individual identification database is to estimate the probability of false matches based on the degree of population substructuring and inbreeding [55]. When evaluating the weight of evidence for the origin to an unknown sample, an allele frequency database is required. The allele frequencies are needed to inform about probabilities for a random genotype match. Typically databases are drawn from broad populations as shown in human forensics [56]. Likewise, all the *S*. *platyclados* populations were combined into one broad population database of Malaysia.

The occurrence of genetic structure is most often created by restricted gene flow due to habitat fragmentation or intrinsic low dispersal abilities [57]. The lower *θ* value in Western compared to Eastern Malaysia might suggest the populations within this region have shared a common gene pool or have experienced extensive gene flow in the past. As this species are located at higher altitude and thus may always experience a strong blowing wind environment which may cause high dispersal abilities for the insect pollinators or seeds in long distance travelling. Besides the genetic structure, the presence of inbreeding based on the calculated *f* values was also noticed in the database. Therefore, the degrees of population substructuring and inbreeding will be taken into consideration for the generation of individual identification database.

Independence testing for HWE and LE are usually used to validate the STR markers prior to its adoption in forensic studies [29]. Natural populations usually violate HWE to certain degree and causing allele frequencies to change over time and this was observed in the current study where the 15 STR loci used to generate the *S*. *platyclados* database show significant deviation in the independence test. The departures from independence tests can be due to several causes such as population substructuring, inbreeding and presence of null alleles [29, 58, 59]. The current study revealed that the significant deviation from HWE observed in Malaysian database might due to the Wahlund effect caused by population substructuring of the split between Western and Eastern Malaysia. Therefore, the “subpopulation-cum-inbreeding model” [46] that accounts for subpopulation and inbreeding effects, was used to correct for these effects in calculating the profile frequency for Malaysian database. The subpopulation-cum-inbreeding model was also adopted in previous studies of *N*. *heimii* (Tnah *et al*., 2010) and *G*. *bancanus* (Ng *et al*., 2016a). The adopted model allowed for the estimation of the probability of false matches by evaluating the degree of population substructuring and inbreeding in plants (Balding and Nichols, 1994). The calculated *θ* value of 0.0603 for Malaysia produces *P*_origin_ > *P*_combined_ causing *d* to be positive and this implies the Malaysian database is non-conservative. In order to ensure the conservativeness of the database with 100% negative *d* value, the *θ* adjustment was required until 0.0800 for the Western Malaysia and 0.2200 for the Eastern Malaysia. After adjustment, the Malaysian database would be feasible to be used to estimate the random match probability of a log with its original stumps. For example, if an illegal *S*. *platyclados* log and stumps of illegal felled trees were found to have originated from Bukit Tapah Forest Reserve, the random match probability of the suspected log to its original stump could be estimated for evidence used by authority to prosecute the illegal logger in the court.

The level of population differentiation will affect the resolution obtained in most assignment tests which rely on the use of STR markers. The assignment rate in Eastern Malaysia is higher than in Western Malaysia and this might be due to stronger population differentiation in Eastern Malaysia (*θ* = 0.1064) compared to Western Malaysia (*θ* = 0.0333). The level of differentiation is given by the coancestry coefficient *θ* value, which is the proportion of total genetic variation attributable to the genetic differences among populations. Our results show that in situations of weak differentiation among source populations, there is reduced ability to assign a specific individual to a given population with significant statistical power. In tropical canopy trees, wind dispersal has the potential to move some seeds over long distances, thus reducing population differentiation [60]. However, this is unlikely to explain the pattern in Eastern Malaysia, where sampling density will have driven the value of differentiation up compared to Western Malaysia. Although *Shorea* species are mostly pollinated by thrips, a minute and energetically limited insect [61]; pollination between trees with distance as far as 240 m was possible by the aerial dispersal of thrips mediated by air current [62]. The *S*. *platyclados* trees are located at high elevation around 1000 m and the air current could have come from the lower ravines or high hillsides to transport the thrips to complete the pollination work. In addition, the seeds produced by *S*. *platyclados* may be moved by wind especially at the high elevation where the species commonly found. The distribution of species sampled in Western Malaysia was found in the continuous mountain range running from the north (Thailand border) southwards to Negeri Sembilan. This continuous topography might have acted as natural assistance for the dispersal of the seeds or pollen of the species.

Both the population and individual identification databases were used to infer the origin of *S*. *platyclados* planted in FRIM campus. The population identification database was able to trace the geographic origin of the 75% of the tested *S*. *platyclados* to Western Malaysian origins based on shared haplotypes, seemingly unique to this region. Although haplotype H2 is found in both Western and Eastern Malaysia, the chances of transporting the seed from Eastern Malaysia to Western Malaysia are small due to the constraints of costs involved for logistic arrangement between the two regions. Subsequently, the individual identification database was used to assign the origin of these individuals to four populations in the northwest area of Western Malaysia. This result has helped to test the efficiency of the timber tracking method developed and subsequently for documentation of tree origin in FRIM campus. As such, this study provides an additional utility to the existing timber tracking system and assistance to the Forest Department in controlling illegal logging as well as to manage the sustainability of this valuable timber species.

For future studies, experiments should be carried out to test the possibility of extracting DNA from dry wood. This is important as the suspected illegal wood sample might be a log that already dried up or processed in the form of wood products. In order to meet the challenges of a forensic tool that can be used to provide evidence suitable for presentation in a court of law, the methods used in this study could be improved by subjecting them to the extensive validation guidelines developed by the Scientific Working Group on DNA Analysis Methods [63]; as well as the recommendations proposed by the International Society for Forensic Genetics Commission on the use of non-human DNA samples in the criminal justice system [64]. Moreover, by cross reference to the Best Practice Guide for Forensic Timber Identification developed by United Nations Office on Drugs and Crime, the forensic data could be ensured to be credible and admissible in court [22]. Therefore, the use of the method is scientifically verified and at the same time meeting the requirements of law enforcement operations.

## Conclusions

*S*. *platyclados* is an important Dark Red Meranti wood which is traded in the international timber market. The present study has explored the abilities and potentials of DNA based techniques for tracing timber to its origin source. It is one of the examples that showing the molecular markers are applicable in timber tracking system. Moreover, the unique properties of DNA present in wood can provide unambiguous validation of the chain-of-custody documentation. The databases could be used to track logs from the stump to the end user and can be used to certify that a wood product is genuinely derived from planted or sustainably managed forest. Thus, for the timber tracking system presented here, we hope with additional efforts to fill the gaps recognized, this study can contribute to forensic applications and ensure the sustainable utilization of the species into the future.

## Supporting information

S1 Fig Graph of Delta *K* showing *K* = 2 as the most probable number of genetic clusters. (DOCX)

S1 Table Seven chloroplast DNA primers pair used in the study. (DOCX)

S2 Table Variable sites (base substitutions and indels) across the seven chloroplast intergenic regions (trnT-trnL, trnS-trnG, atpB-rbcL, petG-trnP, trnG-atpA, psbM-trnD and trnG-rps14) used to delimit the *Shorea platyclados* in the population identification database. (DOCX)

S3 Table Allele frequencies for each of the 15 STR markers used to delimit the Malaysian Database of *Shorea platyclados*. Minimum allele frequencies were adjusted for alleles falling below the (5/2n = 0.0024) threshold.
(DOCX)

S4 Table The evaluations of Hardy-Weinberg equilibrium (HWE) and linkage equilibrium on the 15 STR loci of *Shorea platyclados* in each population. (DOCX)
