# Supplementary figures and images for: Geographic origin and individual assignment of Shorea platyclados (Dipterocarpaceae) for forensic identification
Source: PLoS One. 2017 Apr 21;12(4):e0176158. doi: 10.1371/journal.pone.0176158 (PMC5400268; doi:10.1371/journal.pone.0176158)

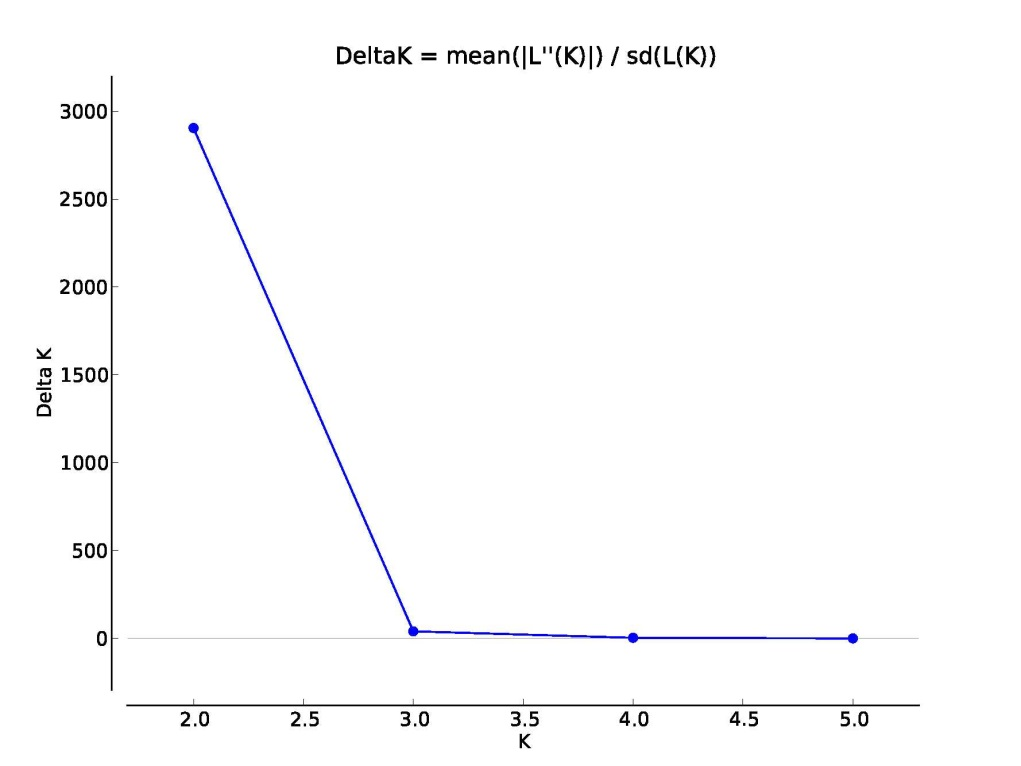


**S1 Fig. Graph of Delta *K* showing *K* = 2 as the most probable number of genetic clusters.**

Supplement: S1 Fig — (DOCX) [file pone.0176158.s001.docx]
