# Supplementary material for: Geographic origin and individual assignment of Shorea platyclados (Dipterocarpaceae) for forensic identification
Source: PLoS One. 2017 Apr 21;12(4):e0176158. doi: 10.1371/journal.pone.0176158 (PMC5400268; doi:10.1371/journal.pone.0176158)
Supplement: S1 Table — (DOCX) [file pone.0176158.s002.docx]

**S1 Table. Seven chloroplast DNA primers pair used in the study.**

| Intergenic  spacer | Primer name | Primer sequence (5’ – 3’) | References |
| --- | --- | --- | --- |
| *trn*T-*trn*L | ucp-a | F:cattacaaatgcgatgctct | [1] |
|  | ucp-b | R:tctaccgatttcgccatatc | [1] |
| *trn*S-*trn*G | *trn*S(GCU) | F:GCCGCTTTAGTCCACTCAGC | [2] |
|  | *trn*G(UCC) | R:GAACGAATCACACTTTTACCAC | [2] |
| *atp*B-*rbc*L | *atp*B | F:CRGGTTGAGGAGTTACTCG | [3] |
|  | *rbc*L | R:GACCRGAAGTAGTAGGATT | [3] |
| *pet*G-*trn*P | *pet*G | F:GGTCTAATTCCTATAACTTTGGC | [4] |
|  | *trn*P | R:GGGATGTGGCGCAGCTTGG | [4] |
| *trn*G-*atp*A | *trn*G1f | F:cgggttcgattcccgctacc | [5] |
|  | *atp*A-r1 | R:CCCTTTTGAAAGAAGCTATTCAGG | [5] |
| *psb*M-*trn*D | *psb*M-f2 | F:ataaatgcaagaatatttacttcc | [5] |
|  | *trn*D-M | R:gggattgtagttcaattggt | [6] |
| *trn*G-*rps*14 | *trn*G | F:TTGCCAAGGAGAAGATGCG | [7] |
|  | *rps*14 | R:ctatccggacacatacttcg | [7] |

**References**

1. Taberlet P, Gielly L, Pautou G, Bouvet J. Universal primers for amplification of three non-coding regions of chloroplast DNA. Plant Mol Biol. 1991;17:1105-9.

2. Hamilton MB. Four primer pairs for the amplification of chloroplast intergenic regions with intraspecific variation. Mol Ecol. 1999;8(3):521-3. Epub 1999/04/13. PubMed PMID: 10199016.

3. Chung JD, Lin TP, Chen YL, Cheng YP, Hwang SY. Phylogeographic study reveals the origin and evolutionary history of a Rhododendron species complex in Taiwan. Mol Phylogenet Evol. 2007;42(1):14-24. Epub 2006/10/31. doi: 10.1016/j.ympev.2006.06.027. PubMed PMID: 17070712.

4. Huang S-F, Hwang S-Y, Wang J-C, Lin T-P. Phylogeography of Trochodendron aralioides (Trochodendraceae) in Taiwan and its adjacent areas. J Biogeogr. 2004;31(8):1251-9. doi: 10.1111/j.1365-2699.2004.01082.x.

5. Heinze B. A database of PCR primers for the chloroplast genomes of higher plants. Plant Methods. 2007;3(1):1-7. doi: 10.1186/1746-4811-3-4.

6. Demesure B, Sodzi N, Petit RJ. A set of universal primers for amplification of polymorphic non-coding regions of mitochondrial and chloroplast DNA in plants. Mol Ecol. 1995;4(1):129-31. Epub 1995/02/01. PubMed PMID: 7711952.

7. Doyle JJ, Davis JI, Soreng RJ, Garvin D, Anderson MJ. Chloroplast DNA inversions and the origin of the grass family (Poaceae). Proc Natl Acad Sci U S A. 1992;89(16):7722-6. Epub 1992/08/15. PubMed PMID: 1502190; PubMed Central PMCID: PMCPmc49783.
