# Supplementary material for: Geographic origin and individual assignment of Shorea platyclados (Dipterocarpaceae) for forensic identification
Source: PLoS One. 2017 Apr 21;12(4):e0176158. doi: 10.1371/journal.pone.0176158 (PMC5400268; doi:10.1371/journal.pone.0176158)
Supplement: S2 Table — (DOCX) [file pone.0176158.s003.docx]

**S2 Table. Variable sites (base substitutions and indels) across the seven chloroplast intergenic regions (*trn*T-*trn*L, *trn*S-*trn*G, *atp*B-*rbc*L, *pet*G-*trn*P, *trn*G-*atp*A, *psb*M-*trn*D and *trn*G-*rps*14) used to delimit the *Shorea platyclados* in the population identification database.**

| Haplotype | *trn*T-*trn*L | |  | *trn*S-*trn*G | | | | | | | |  | *atp*B-*rbc*L | | | | |  | *pet*G-*trn*P | | |  | *trn*G-*atp*A | | | | |  | *psb*M-*trn*D | | | | | | | | | | |  | *trn*G-*rps*14 | | | | |
| --- | --- | --- | --- | --- | --- | --- | --- | --- | --- | --- | --- | --- | --- | --- | --- | --- | --- | --- | --- | --- | --- | --- | --- | --- | --- | --- | --- | --- | --- | --- | --- | --- | --- | --- | --- | --- | --- | --- | --- | --- | --- | --- | --- | --- | --- |
|  | 92 | 201 |  | 161 | 208 | 217 | 231 | 259-260 | 261-265 | 266-268 | 269 |  | 71 | 200 | 282 | 389 | 398 |  | 46 | 102 | 131-132 |  | 27-32 | 80 | 140 | 251 | 323 |  | 66 | 159 | 205 | 258 | 279 | 319 | 377 | 441 | 490 | 519 | 550 |  | 11 | 60 | 93 | 98-101 | 319 |
|  |  |  |  |  |  |  |  |  |  |  |  |  |  |  |  |  |  |  |  |  |  |  |  |  |  |  |  |  |  |  |  |  |  |  |  |  |  |  |  |  |  |  |  |  |  |
| H1 | T | G |  | G | T | A | G | CA | TAAAT | TAA | A |  | C | C | T | C | C |  | A | C | AG |  | ATTAAT | G | C | C | T |  | G | - | A | G | C | C | C | C | G | C | T |  | G | C | G | ---- | G |
| H2 | C | G |  | G | G | A | G | CA | TAAAT | TAA | A |  | C | C | T | C | C |  | A | C | AG |  | ATTAAT | G | C | A | T |  | G | - | A | G | C | C | C | C | A | C | T |  | G | C | G | GTCT | G |
| H3 | T | C |  | G | T | A | G | CA | TAAAT | TAA | A |  | C | C | T | C | C |  | A | C | AG |  | ATTAAT | G | C | C | T |  | G | - | A | G | C | C | C | C | G | C | T |  | G | C | G | ---- | G |
| H4 | T | G |  | G | T | A | G | CA | TAAAT | TAA | A |  | C | C | T | C | C |  | A | C | AG |  | ------ | G | C | C | T |  | G | - | A | G | C | C | C | C | G | C | T |  | G | C | G | ---- | G |
| H5 | T | G |  | G | T | A | G | -- | ----- | --- | - |  | C | C | T | C | C |  | A | C | AG |  | ATTAAT | G | C | C | T |  | G | - | A | G | C | C | C | C | G | C | T |  | G | C | G | ---- | G |
| H6 | T | G |  | G | T | A | G | CA | TAAAT | TAA | A |  | C | C | T | C | C |  | A | C | AG |  | ATTAAT | G | C | C | T |  | G | - | A | G | C | G | C | C | G | C | T |  | G | C | G | ---- | G |
| H7 | T | G |  | G | T | A | G | CA | TAAAT | TAA | A |  | C | C | T | C | C |  | A | C | AG |  | ATTAAT | G | C | C | T |  | G | - | A | G | C | C | C | C | G | C | A |  | G | C | G | ---- | G |
| H8 | C | G |  | G | G | A | G | CA | TAAAT | TAA | A |  | C | C | G | C | C |  | A | C | -- |  | ATTAAT | G | C | A | T |  | G | - | A | G | C | C | C | C | A | C | T |  | G | C | G | GTCT | G |
| H9 | C | G |  | G | G | C | G | CA | TAAAT | TAA | A |  | C | C | T | C | C |  | A | C | AG |  | ATTAAT | G | C | A | T |  | G | - | A | G | C | C | C | C | G | C | T |  | G | C | G | GTCT | G |
| H10 | C | G |  | G | G | A | G | CA | TAAAT | TAA | A |  | C | C | G | C | C |  | A | C | AG |  | ATTAAT | G | C | A | T |  | G | - | A | G | C | C | C | C | A | C | T |  | G | C | G | GTCT | G |
| H11 | C | G |  | G | G | A | G | CA | TAAAT | TAA | A |  | C | C | T | C | C |  | A | A | AG |  | ATTAAT | G | C | A | T |  | G | - | A | G | C | C | C | C | A | A | T |  | C | C | G | GTCT | G |
| H12 | C | G |  | G | G | C | G | CA | TAAAT | TAA | A |  | C | C | T | C | C |  | A | C | AG |  | ATTAAT | G | C | A | T |  | G | - | A | G | C | C | C | C | A | C | T |  | G | C | G | GTCT | G |
| H13 | T | G |  | G | T | A | G | CA | TAAAT | TAA | A |  | C | C | T | T | C |  | A | C | AG |  | ATTAAT | G | C | C | T |  | G | - | A | G | C | C | C | C | G | C | T |  | G | C | G | ---- | G |
| H14 | C | G |  | G | G | A | G | CA | TAAAT | TAA | A |  | C | C | T | C | C |  | A | A | AG |  | ATTAAT | G | C | A | T |  | A | - | A | G | C | C | C | A | A | C | T |  | G | C | G | GTCT | G |
| H15 | C | G |  | G | G | A | G | CA | TAAAT | TAA | A |  | C | C | T | C | C |  | A | C | AG |  | ATTAAT | G | C | A | T |  | G | - | A | G | G | C | C | C | A | C | T |  | G | C | G | GTCT | G |
| H16 | C | G |  | G | G | A | G | CA | TAAAT | TAA | A |  | C | C | T | C | C |  | A | C | AG |  | ATTAAT | G | C | A | T |  | G | A | A | G | C | C | C | C | A | C | T |  | G | C | G | GTCT | G |
| H17 | C | G |  | G | G | A | G | CA | TAAAT | TAA | A |  | C | C | T | C | C |  | A | A | AG |  | ATTAAT | G | C | A | T |  | G | - | A | G | C | C | C | C | A | C | T |  | G | C | G | GTCT | G |
| H18 | T | G |  | G | G | A | G | CA | TAAAT | TAA | A |  | C | C | T | C | C |  | A | C | AG |  | ATTAAT | G | C | A | T |  | G | - | A | G | C | C | C | C | A | C | T |  | G | C | G | GTCT | G |
| H19 | T | G |  | - | T | A | G | CA | TAAAT | TAA | A |  | C | C | T | C | C |  | A | C | AG |  | ------ | G | C | C | T |  | G | - | A | G | C | C | C | C | G | C | T |  | G | C | G | ---- | G |
| H20 | C | G |  | G | T | A | C | CA | TAAAT | TAA | A |  | C | C | T | C | C |  | A | C | AG |  | ATTAAT | G | - | A | A |  | G | - | T | G | C | C | C | C | G | C | T |  | G | C | G | GTCT | T |
| H21 | C | G |  | G | T | A | G | CA | TAAAT | TAA | A |  | A | A | T | C | T |  | A | C | AG |  | ATTAAT | G | A | A | A |  | G | - | T | G | C | C | C | C | G | C | T |  | G | C | G | GTCT | T |
| H22 | C | G |  | G | T | A | G | CA | TAAAT | TAA | A |  | C | C | T | C | C |  | A | C | AG |  | ATTAAT | G | C | A | T |  | G | - | A | G | C | C | C | C | G | C | T |  | G | C | G | ---- | G |
| H23 | T | G |  | G | T | A | G | -- | TAAAT | --- | A |  | C | C | T | C | C |  | A | C | AG |  | ATTAAT | G | C | C | T |  | G | - | A | G | C | C | C | C | G | C | T |  | G | C | G | ---- | G |
| H24 | T | G |  | G | T | A | G | CA | TAAAT | TAA | A |  | C | C | T | C | C |  | A | C | AG |  | ATTAAT | C | C | C | T |  | G | - | A | G | C | C | C | C | G | C | T |  | G | C | G | ---- | G |
| H25 | C | G |  | G | G | C | G | CA | TAAAT | TAA | A |  | C | C | T | C | C |  | A | C | AG |  | ATTAAT | G | C | A | T |  | G | - | A | G | C | C | C | C | G | C | T |  | G | C | T | GTCT | G |
| H26 | C | G |  | G | G | C | G | CA | TAAAT | TAA | A |  | C | C | T | C | C |  | A | C | AG |  | ATTAAT | G | C | A | T |  | G | - | A | G | C | C | G | C | A | C | T |  | G | C | G | GTCT | G |
| H27 | T | G |  | G | T | A | G | CA | TAAAT | TAA | A |  | C | C | T | C | C |  | C | C | AG |  | ATTAAT | G | C | C | T |  | G | - | A | G | C | C | C | C | G | C | T |  | G | C | G | ---- | G |
| H28 | T | G |  | G | T | A | G | CA | TAAAT | TAA | A |  | C | C | T | C | C |  | A | C | AG |  | ATTAAT | G | C | C | T |  | G | - | A | A | C | C | C | C | G | C | T |  | G | C | G | ---- | G |
| H29 | T | G |  | G | T | A | G | CA | TAAAT | TAA | A |  | C | C | T | C | C |  | A | C | AG |  | ATTAAT | G | C | C | T |  | G | - | A | G | C | C | C | C | G | C | T |  | G | T | G | ---- | G |
|  |  |  |  |  |  |  |  |  |  |  |  |  |  |  |  |  |  |  |  |  |  |  |  |  |  |  |  |  |  |  |  |  |  |  |  |  |  |  |  |  |  |  |  |  |  |
