# Supplementary material for: Geographic origin and individual assignment of Shorea platyclados (Dipterocarpaceae) for forensic identification
Source: PLoS One. 2017 Apr 21;12(4):e0176158. doi: 10.1371/journal.pone.0176158 (PMC5400268; doi:10.1371/journal.pone.0176158)
Supplement: S3 Table — Minimum allele frequencies were adjusted for alleles falling below the (5/2n = 0.0024) threshold. (DOCX) [file pone.0176158.s004.docx]

**S3 Table. Allele frequencies for each of the 15 STR markers used to delimit the Malaysian Database of *Shorea platyclados*.** Minimum allele frequencies were adjusted for alleles falling below the (5/2n = 0.0024) threshold.

| Allele size - frequency | | | | | | | | | | | | | | |
| --- | --- | --- | --- | --- | --- | --- | --- | --- | --- | --- | --- | --- | --- | --- |
| *Spl*003 | *Spl*529 | *Spl*599 | *Spl*600 | *Spl*629 | *Spl*667 | *Spl*676 | *Spl*690 | *Spl*763 | *Spl*764 | *Spl*834 | *Spl*845 | *Spl*855 | *Spl*858 | *Spl*863 |
| 97 - 0.0005 | 88 - 0.0024 | 219 - 0.0355 | 129 - 0.0024 | 202 - 0.0005 | 150 - 0.0010 | 130 - 0.0005 | 107 - 0.0010 | 172 - 0.0034 | 149 - 0.0267 | 182 - 0.0005 | 136 - 0.0015 | 193 - 0.0010 | 139 - 0.0010 | 109 - 0.0010 |
| 99 - 0.0044 | 90 - 0.0029 | 221 - 0.3959 | 130 - 0.0005 | 204 - 0.0005 | 152 - 0.1830 | 131 - 0.0044 | 124 - 0.0024 | 174 - 0.0087 | 151 - 0.0015 | 184 - 0.0005 | 138 - 0.0034 | 197 - 0.0102 | 142 - 0.0019 | 127 - 0.0024 |
| 100 - 0.0005 | 92 - 0.5490 | 222 - 0.0029 | 132 - 0.0058 | 206 - 0.0005 | 155 - 0.0020 | 133 - 0.0058 | 127 - 0.0015 | 176 - 0.0107 | 153 - 0.0005 | 186 - 0.0736 | 142 - 0.0005 | 199 - 0.0039 | 144 - 0.0005 | 129 - 0.0024 |
| 102 - 0.0015 | 94 - 0.3375 | 223 - 0.2062 | 134 - 0.0034 | 208 - 0.0097 | 156 - 0.0235 | 136 - 0.0165 | 129 - 0.5567 | 178 - 0.0048 | 155 - 0.0005 | 188 - 0.1497 | 144 - 0.0005 | 200 - 0.5383 | 146 - 0.0741 | 131 - 0.5563 |
| 103 - 0.0113 | 96 - 0.0107 | 225 - 0.0277 | 136 - 0.0223 | 209 - 0.0010 | 159 - 0.0029 | 138 - 0.1832 | 131 - 0.3288 | 180 - 0.0019 | 157 - 0.0306 | 190 - 0.1090 | 145 - 0.0005 | 202 - 0.2582 | 150 - 0.0005 | 133 - 0.3283 |
| 105 - 0.0024 | 98 - 0.0039 | 226 - 0.0005 | 138 - 0.5015 | 210 - 0.0024 | 160 - 0.0039 | 139 - 0.0019 | 132 - 0.0010 | 182 - 0.1216 | 158 - 0.0272 | 192 - 0.0087 | 146 - 0.0005 | 208 - 0.0029 | 152 - 0.0015 | 134 - 0.0015 |
| 107 - 0.1169 | 100 - 0.0107 | 227 - 0.1240 | 140 - 0.1982 | 211 - 0.0015 | 161 - 0.0851 | 140 - 0.0121 | 133 - 0.0112 | 184 - 0.4792 | 159 - 0.0005 | 194 - 0.0678 | 147 - 0.0215 | 216 - 0.0068 | 154 - 0.0523 | 135 - 0.0107 |
| 109 - 0.0685 | 103 - 0.0126 | 229 - 0.1732 | 142 - 0.2495 | 212 - 0.0160 | 162 - 0.0024 | 141 - 0.0039 | 135 - 0.0044 | 186 - 0.2422 | 161 - 0.0558 | 196 - 0.0199 | 148 - 0.0005 | 218 - 0.0838 | 157 - 0.2025 | 137 - 0.0044 |
| 111 - 0.3009 | 105 - 0.0039 | 231 - 0.0175 | 144 - 0.0044 | 214 - 0.1329 | 163 - 0.1062 | 142 - 0.0039 | 137 - 0.0107 | 188 - 0.0775 | 164 - 0.5534 | 198 - 0.0029 | 149 - 0.0420 | 220 - 0.0659 | 158 - 0.0005 | 139 - 0.0107 |
| 114 - 0.2412 | 107 - 0.0092 | 233 - 0.0088 | 145 - 0.0058 | 216 - 0.0276 | 165 - 0.2485 | 143 - 0.0024 | 139 - 0.0126 | 191 - 0.0136 | 166 - 0.0209 | 200 - 0.0073 | 151 - 0.0010 | 222 - 0.0116 | 159 - 0.2524 | 141 - 0.0126 |
| 116 - 0.0127 | 109 - 0.0412 | 237 - 0.0063 | 147 - 0.0053 | 217 - 0.0005 | 167 - 0.0220 | 144 - 0.0224 | 142 - 0.0039 | 193 - 0.0247 | 168 - 0.1801 | 202 - 0.2762 | 152 - 0.0093 | 224 - 0.0019 | 161 - 0.1303 | 143 - 0.0039 |
| 118 - 0.0010 | 111 - 0.0034 | 240 - 0.0010 | 151 - 0.0010 | 218 - 0.5936 | 168 - 0.0029 | 145 - 0.0141 | 144 - 0.0082 | 195 - 0.0039 | 170 - 0.0636 | 204 - 0.1473 | 153 - 0.0015 | 230 - 0.0010 | 163 - 0.0596 | 145 - 0.0082 |
| 120 - 0.0024 | 113 - 0.0112 | 242 - 0.0005 |  | 219 - 0.0010 | 169 - 0.0440 | 146 - 0.0899 | 146 - 0.0407 | 197 - 0.0005 | 173 - 0.0015 | 206 - 0.1226 | 154 - 0.0117 | 235 - 0.0005 | 165 - 0.0368 | 147 - 0.0407 |
| 122 - 0.1742 | 120 - 0.0005 |  |  | 220 - 0.1227 | 171 - 0.0113 | 147 - 0.0092 | 148 - 0.0034 | 199 - 0.0010 | 174 - 0.0068 | 208 - 0.0097 | 156 - 0.0024 | 237 - 0.0024 | 167 - 0.0208 | 149 - 0.0034 |
| 124 - 0.0563 | 122 - 0.0005 |  |  | 222 - 0.0252 | 172 - 0.0005 | 148 - 0.0097 | 149 - 0.0010 | 201 - 0.0029 | 176 - 0.0131 | 210 - 0.0019 | 158 - 0.0766 | 239 - 0.0048 | 169 - 0.0518 | 151 - 0.0121 |
| 130 - 0.0005 | 124 - 0.0005 |  |  | 224 - 0.0519 | 174 - 0.0044 | 149 - 0.0005 | 150 - 0.0112 | 207 - 0.0024 | 177 - 0.0005 | 214 - 0.0005 | 159 - 0.0005 | 241 - 0.0015 | 171 - 0.0252 | 159 - 0.0005 |
| 132 - 0.0049 |  |  |  | 225 - 0.0019 | 176 - 0.0196 | 150 - 0.1239 | 158 - 0.0005 | 209 - 0.0005 | 178 - 0.0068 | 220 - 0.0019 | 160 - 0.1561 | 243 - 0.0029 | 173 - 0.0300 | 163 - 0.0005 |
|  |  |  |  | 226 - 0.0015 | 178 - 0.0504 | 151 - 0.0112 | 162 - 0.0005 | 211 - 0.0005 | 179 - 0.0005 |  | 162 - 0.3590 | 245 - 0.0019 | 175 - 0.0097 | 167 - 0.0005 |
|  |  |  |  | 227 - 0.0010 | 180 - 0.1233 | 152 - 0.1331 | 166 - 0.0005 |  | 180 - 0.0019 |  | 164 - 0.1771 | 251 - 0.0005 | 177 - 0.0150 |  |
|  |  |  |  | 229 - 0.0073 | 182 - 0.0445 | 153 - 0.0019 |  |  | 182 - 0.0053 |  | 166 - 0.0795 |  | 180 - 0.0150 |  |
|  |  |  |  | 235 - 0.0010 | 184 - 0.0064 | 154 - 0.1093 |  |  | 185 - 0.0005 |  | 168 - 0.0332 |  | 182 - 0.0092 |  |
|  |  |  |  |  | 186 - 0.0078 | 155 - 0.0019 |  |  | 186 - 0.0015 |  | 170 - 0.0088 |  | 184 - 0.0019 |  |
|  |  |  |  |  | 189 - 0.0020 | 156 - 0.1059 |  |  |  |  | 172 - 0.0102 |  | 186 - 0.0029 |  |
|  |  |  |  |  | 191 - 0.0005 | 158 - 0.0345 |  |  |  |  | 175 - 0.0005 |  | 188 - 0.0029 |  |
|  |  |  |  |  | 193 - 0.0005 | 160 - 0.0364 |  |  |  |  | 176 - 0.0005 |  | 190 - 0.0015 |  |
|  |  |  |  |  | 195 - 0.0015 | 162 - 0.0301 |  |  |  |  | 177 - 0.0005 |  |  |  |
|  |  |  |  |  |  | 164 - 0.0146 |  |  |  |  | 187 - 0.0005 |  |  |  |
|  |  |  |  |  |  | 167 - 0.0024 |  |  |  |  | 191 - 0.0005 |  |  |  |
|  |  |  |  |  |  | 169 - 0.0044 |  |  |  |  |  |  |  |  |
|  |  |  |  |  |  | 171 - 0.0053 |  |  |  |  |  |  |  |  |
|  |  |  |  |  |  | 173 - 0.0044 |  |  |  |  |  |  |  |  |
