# Supplementary material for: Geographic origin and individual assignment of Shorea platyclados (Dipterocarpaceae) for forensic identification
Source: PLoS One. 2017 Apr 21;12(4):e0176158. doi: 10.1371/journal.pone.0176158 (PMC5400268; doi:10.1371/journal.pone.0176158)
Supplement: S4 Table — (DOCX) [file pone.0176158.s005.docx]

**S4 Table. The evaluations of Hardy-Weinberg equilibrium (HWE) and linkage equilibrium on the 15 STR loci of *Shorea platyclados* in each population.**

| Population | Code | Significant deviations from HWE  after Bonferroni adjustment (*P* < 0.0033) | | Significant pairwise after Bonferroni adjustment (*P* < 0.00048) |  |
| --- | --- | --- | --- | --- | --- |
|  |  |  |  |  |  |
| Petuang | 1 | nil | | 2.9% | |
| Tembat | 2 | *Spl*600, *Spl*629 | | 4.8% |  |
| Hulu Terengganu | 3 | *Spl*600, *Spl*763, *Spl*855 | | 8.6% |  |
| Gunung Basur | 4 | nil | | 2.9% |  |
| Balah | 5 | nil | | 2.9% |  |
| Gunung Stong | 6 | nil | | 2.9% |  |
| Sungai Betis | 7 | nil | | 2.9% |  |
| Gunung Rabong | 8 | nil | | 2.9% |  |
| Belum | 9 | *Spl*529 | | 2.9% |  |
| Temenggor | 10 | nil | | 1.9% |  |
| Piah | 11 | nil | | 2.9% |  |
| Bintang Hijau | 12 | nil | | 2.9% |  |
| Bukit Larut | 13 | nil | | 2.9% |  |
| Bubu | 14 | nil | | 2.9% |  |
| Bukit Kinta | 15 | nil | | 2.9% |  |
| Bukit Tapah | 16 | nil | | 0% |  |
| Ulu Jelai | 17 | nil | | 0% |  |
| Fraser | 18 | nil | | 5.7% |  |
| Awana | 19 | nil | | 0% |  |
| Gunung Bunga Buah | 20 | nil | | 2.9% |  |
| Bukit Tinggi | 21 | nil | | 3.8% |  |
| Lentang | 22 | *Spl*834 | | 7.6% |  |
| Semangkok | 23 | nil | | 2.9% |  |
| Berembun | 24 | nil | | 2.9% |  |
| Nanga Amang | 25 | nil | | 2.9% |  |
| Putai | 26 | *Spl*003, *Spl*600, *Spl*763, *Spl*764, *Spl*855 | | 19.0% |  |
| Rafflesia | 27 | *Spl*003 | | 2.9% |  |
